# Temporal Lobe Necrosis Following Radiotherapy in Nasopharyngeal Carcinoma: New Insight Into the Management

**DOI:** 10.3389/fonc.2020.593487

**Published:** 2021-01-21

**Authors:** Xin Zhou, Peiyao Liu, Xiaoshen Wang

**Affiliations:** ^1^ Department of Radiation Oncology, Fudan University Shanghai Cancer Center, Shanghai, China; ^2^ Department of Oncology, Shanghai Medical College, Fudan University, Shanghai, China; ^3^ Department of Radiation Oncology, Eye and ENT Hospital, Fudan University, Shanghai, China

**Keywords:** cerebral radiation necrosis, pathophysiology, bevacizumab, monosialotetrahexosylganglioside, nerve growth factor

## Abstract

Cerebral radiation necrosis (CRN) is one of the most prominent sequelae following radiation therapy for nasopharyngeal carcinoma (NPC), which might have devastating effects on patients’ quality of life (QOL). Advances in histopathology and neuro-radiology have shed light on the management of CRN more comprehensively, yet effective therapeutic interventions are still lacking. CRN was once regarded as progressive and irreversible, however, in the past 20 years, with the application of intensity-modulated radiation therapy (IMRT), both the incidence and severity of CRN have declined. In addition, newly developed medical agents including bevacizumab-a humanized monoclonal antibody against vascular endothelial growth factor (VEGF), nerve growth factor (NGF), monosialotetrahexosylganglioside (GM1), etc., have shown great potency in successfully reversing radiation-induced CRN. As temporal lobes are most frequently compromised in NPC patients, this review will summarize the state-of-the-art progress regarding the incidence, pathophysiology, prevention, treatment, and prognosis of temporal lobe necrosis (TLN) after IMRT in NPC.

## Introduction

Nasopharyngeal carcinoma (NPC) constitutes the largest proportion of head and neck malignancies in China and Southeast-Asia, and radiation therapy (RT) is the mainstay treatment for non-metastatic cases. In the past decade, advances in intensity-modulated radiation therapy (IMRT) have allowed for improved spatial dose distribution, hence better preserving organs at risk (OARs). However, due to the anatomical proximity between nasopharynx and cerebrum, cerebral radiation necrosis (CRN) remains conspicuous as a late complication following IMRT. Particularly, for those with skull-base or intracranial invasion, overlap with radiation target volumes tends to generate dosimetric “hot spots” in temporal lobes (TLs) even with IMRT (1), making temporal lobe necrosis (TLN) a relatively common form of CRN in NPC. As is frequently accompanied with symptomatic abnormalities such as lethargy, dizziness, debilitation, emotional disorders, cognitive dysfunction, and even epileptic attacks, TLN may significantly impair survivors’ quality of life (QOL) (2). Accumulating evidence have suggested the etiology and pathogenesis of CRN, nevertheless, many questions remain unanswered regarding its management. This review, with emphasis on therapeutic perspective, will focus on CRN, especially TLN after radiotherapy for NPC.

## Incidence and Risk Factors of Temporal Lobe Necrosis

TLN is a joint effect of genetic, clinical, and RT-related factors (**Table 1**) (3–10). Radiation techniques and RT parameters constitute the most critical part of RT-related factors. RT parameters, including dose fractionation, total radiation dose, irradiated volume, etc., were thought to most profoundly affect the development of TLN. Generally, increased total RT dose or larger dose per fraction is associated with escalating risk of TLN and shortened latency (11). The evolution in RT technique have also led to a fundamental change in CRN incidence. Back in the era of two-dimensional conventional radiotherapy (2D-CRT), with different fractionation strategy, the incidence of TLN varied from 1.6 to 22% at an interval of 9 months to 16 years after treatment (12, 13). Lee et al. reported that a total dose of 64 Gy in 32 fractions would lead to 5% necrotic rate in 10 years (9). However, with IMRT widely used in NPC, the rate of TLN tended to decline in long-term survivors. Zhou et al. retrospectively reviewed 1,276 NPC patients and found that IMRT yielded a significantly decreased 5-year actuarial incidence of TLN (16.0 *vs.* 34.9%, P < 0.001) (4). Another study, through prospectively randomization, also found that NPC patients receiving IMRT had lower rate of TLN (13.1 *vs.* 21%) (10). Meanwhile, in comparison to the commonly seen bilateral TLN lesions in 2D-CRT era, IMRT-induced TLN mostly occurred ipsilaterally with reduced size (14). These improvements, to a large extent, might be attributed to the dosimetric advantage of IMRT in sparing temporal lobes by reducing regions with high-dose irradiation (15).

**Table 1:** Risk factors of radiation-induced temporal lobe injury in nasopharyngeal carcinoma.

Risk factors	Authors	Study type	Enrolled patients	RT technique	Results
Genetic susceptibility	Wang et al. (3)	Prospective, observational	Discovery stage: 1,082; Validation stage I: 1,119; Validation stage II: 741	2D-RT; IMRT	Minor alleles at rs162171 or rs17111237 are related to higher risk of TLI (per allele HR, 1.46 and 1.45).
Tumor stage	Zhou et al. (4)	Retrospective	1,276, firstly diagnosed NPC	2D-RT; IMRT	T classification is an independent predictor of TLI (T3-4 *vs.* T1-2 HR, 2.777).
Chemotherapy	Zeng et al. (5)	Retrospective	789, firstly diagnosed NPC	IMRT	Chemoradiation *vs.* RT: 5-year actuarial incidence of TLN, 10.1 *vs.* 1.9% (HR, 2.58, P = 0.030).
Targeted therapy	Niu et al. (6)	Retrospective	33, firstly diagnosed NPC	IMRT	Concurrent cetuximab plus IMRT with/out chemotherapy: unexpectedly high TLN rate, 21.2%.
	Ng et al. (7)	Prospective, single-arm, phase II	33, recurrent T3-4 NPC	IMRT	Concurrent bio-chemoradiation with cetuximab: high TLN rate, 30.8%.
Fractional dose	Teo et al. (8)	Retrospective	159, firstly diagnosed NPC	2D-RT	Late course HART (1.6 Gy, twice daily) *vs.* conventional fractionation (2.5 Gy daily): TLN rate, 40.2 *vs.* 19.5%.
	Lee et al. (9)	Retrospective	1,008, firstly diagnosed NPC	2D-RT	Hypofractionation (4.2 Gy daily) *vs.* conventional fractionation (2.5 Gy daily): 10-year actuarial incidence of TLN, 18.6 *vs.* 4.6%.
RT technique	Peng et al. (10)	Prospective, randomized, phase III	616, firstly diagnosed NPC	2D-RT; IMRT	2D-CRT *vs.* IMRT: TLN rate, 21.0 *vs.* 13.1%.

NPC, nasopharyngeal carcinoma; 2D-RT, two-dimensional radiotherapy; HART, hyperfractionated accelerated radiotherapy; IMRT, intensity-modulated radiotherapy; TLI, temporal lobe injury; TLN, temporal lobe necrosis; HR, hazard ratio.

Non-RT factors, such as genetic susceptibility, chemotherapy, and targeted therapy, might exacerbate the occurrence of TLN. According to Wang et al., centrosome protein CEP128 links to the maintenance of cell radioresistance, downregulation of CEP128 by genetic variants could remarkably add to the radiation damage of glial cells, and further increase the risk of CRN (3). Ruben et al. reported that post-RT chemotherapy enhanced the hazard of CRN by approximately fivefold in patients with glioma (16). In NPC, chemotherapy was also reported to be an independent risk factor that promoted the 5-year incidence of TLN from 1.9%to 10.1% (5). The impact of targeted agents on TLN is yet uncertain, but some studies have suggested that cetuximab, a monoclonal antibody to epidermal growth factor receptor (EGFR), might confer relatively high risk of TLN when used concurrently with RT in both treatment-naïve and recurrent NPC patients (6, 7). Future work is warranted to specifically illustrate the role of anti-EGFR agents in TLN development as well as the potential biological mechanisms.

## Pathophysiology of Radiation Induced Necrosis to the Brain

RT induced brain injury includes early-phase changes such as acute edema or subacute demyelination, and late changes featured by delayed CRN. While acute edema could be reversed with timely intervention, CRN usually presents with an unpredictable pattern of evolution, bringing more difficulty to the recognition of its pathogenesis and management. Up to now, the mechanisms of CRN development have not been completely understood. The typical pathological presentation of CRN was first described by Lowenberg-Scharenberg et al. as amyloid degeneration in 1950 (17). Subsequent investigations found that CRN was histologically featured by coagulation necrosis in the white matter, presenting fibrinoid necrosis and hyalinization of vessel walls, telangiectasis, dystrophic calcification as well as surrounding inflammation and gliosis (18). Immunohistochemistry further showed expression of hypoxia-inducible factor-1a (HIF-1α), vascular endothelial growth factor (VEGF) and inflammatory cytokines like Interleukin-6 and tumor necrosis factor (TNF)-α in glial cells near necrotic area (19). Based on published literatures, RT-induced cerebral tissue injury is a highly complex process that involves multiple tissue elements (20–22). Three models have been postulated to eventually contribute to the occurrence of CRN: (a) vascular endothelial injury: radiation injury to endothelial cells and following apoptosis provokes massive release of oxygen free radicals, hence inducing upregulation of HIF-1α and VEGF, causing blood-brain-barrier (BBB) disruption, vasogenic edema, platelet and fibrin thrombi formation, vessel occlusion, and ischemic changes. (b) injury to glial/progenitor cells: radiation can directly damage astrocytes, oligodendrocytes, and their progenitors, correspondingly causing hypocellular architectural changes such as BBB breakdown with worsened edema and hypoxia, astrogliosis, and demyelination. The production of VEGF and delayed release of TNF-α by microglia and astrocytes in perinecrotic zone further aggravates this process, eventually forming a vicious cycle. (c) immuno-inflammation induced injury: under radiation stress, lymphocytes and macrophages infiltrate in perivascular and parenchymal spaces, actively secreting inflammatory cytokines; microglia cells are also stimulated and contribute to the inflammatory response, exacerbating BBB permeability defect and hypoxia-induced necrosis (18–22). In general, vascular injury-induced white matter edema occurs as an acute toxicity, followed by glial cell-related subacute demyelination, and eventually evolutes into a delayed phase of brain necrosis (23–25).

## Prevention of Cerebral Radiation Necrosis

Despite the multiple pharmacological efforts to treat CRN, the most pragmatic and cost-effective approach to manage remains prevention. As more dose-volume-histogram (DVH) data being published, consensus has been established that CRN is actually a function of both irradiation dose and volume. For temporal lobes, currently the most widely accepted dose constraint is the recommendation from Radiation Therapy Oncology Group (RTOG) 0225, which confined the maximum dose (Dmax) to lower than 60 Gy and 1% of the temporal lobe volume not exceeding 65 Gy (26). This constraint, however, might be too stringent sometimes, especially in those with locally advanced NPC that locate adjacently or even overlapped with temporal lobes. According to Su et al., no temporal lobe with Dmax <64 Gy had necrosis, but the incidence increases by 2.6% per Gy increment of Dmax once exceeding 64 Gy. They further recommended Dmax <68 Gy as a safe constraint for IMRT plans (27). Zeng et al. reported an escalating 5-year TLN rate from 0.8% in TLs with Dmax <65.77 Gy to 27.1% in those with greater dose (5). Another analysis by Zeng et al. plotted the dose-response curves and estimated the tolerance dose (TD) for the 5% probability of TLI at 62.83 Gy equivalents (28). Kong et al. estimated TD5/5 of TLN was Dmax at 69.0 ± 1.6 Gy and D1cc (maximum dose delivered to a volume of 1 ml) at 62.8 ± 2.2 Gy (29). Wang et al. determined through LASSO (least absolute shrinkage and selection operator) regressions that D0.5cc and D10 were reliable dosimetric predictors of TLN (30). These studies suggested that the maximum dose to TLs might be safely loosened under specific circumstances. Therefore, the 2019 international guideline of RT planning for NPC recommended a stepwise dose constraint for temporal lobes: D0.03cc ≤ 65 Gy for early stage and ≤70 Gy for advanced stage. In the difficulty of balancing TL protection and tumor control, even D0.03cc ≤ 72 Gy could be accepted (31). In another aspect, new concerns were raised regarding the role of volumetric factors in TLN development. Su et al. found that aV_40_ (absolute volume receiving dose over 40 Gy) and rV_40_ (the percentage of V40 in total TL volume) in TLs as independent risk factors for TLN, and further proposed new dose constraints of rV_40_<10% or aV_40_<5 cc to TLs (32). Zhou et al. further investigated the relationship between volumetric factors and the extent of TLN, and drew a conclusion that V_45_ >15.1cc tended to induce larger lesion when TLN happened (33). Therefore, inverse IMRT plans should maximally avoid not only focal “hot spot” dose, but also moderate dose delivered to a large area in TLs. Details of dose constraint recommendation are listed in **Table 2** (1, 27–30, 32–37).

**Table 2:** Dose constraints to prevent TLN in IMRT planning for nasopharyngeal carcinoma.

Authors	Study type	Study objects	Variables	Results	Proposed dose constraints
Sun et al. (1)	Case-controlled	20 patients, 20 pairs of necrotic and normal TLs	D0.5cc	AUC for D0.5cc in predicting TLN = 0.843.	D0.5cc<69 Gy
Su et al. (27)	Cohort, retrospective	251 patients, 43 necrotic and 431 normal TLs	Dmax, D1cc	TLN incidence augmented by 2.6 and 2.5% per Gy for Dmax >64 Gy and D1cc >52 Gy, respectively.	Dmax<68 Gy; D1cc<58 Gy
Zeng et al. (5)	Cohort, retrospective	230 patients, 48 necrotic and 412 normal TLs	Dmax	5-year TLN incidence for TLN: Dmax ≥65.77 Gy, 0.8% Dmax <65.77 Gy, 27.1%	Dmax<65.77 Gy
Zeng et al. (28)	Cohort, retrospective	278 patients, 35 necrotic and 517 normal TLs	D1cc	TD5/5 for D1cc = 62.8 Gy; TD50/5 for D1cc = 77.6 Gy.	D1cc<62.8 Gy
Kong et al. (29)	Cohort, retrospective	132 patients, 42 necrotic and 222 normal TLs	Dmax, D1cc	TD5/5 for Dmax = 69.0 ± 1.6, TD50/5 for Dmax = 82.1 ± 2.4 Gy; TD5/5 for D1cc = 62.8 ± 2.2, TD50/5 for D1cc = 80.9 ± 3.4 Gy.	Dmax<69.0 Gy; D1cc<62.8 Gy
Wang et al. (30)	Cohort, retrospective	749 patients, 79 necrotic and 1419 TLs	D0.5cc, D10	LASSO prediction model with D0.5cc and D10: C-index, 0.685.	NA
Su et al. (32)	Cohort, retrospective	259 patients, 47 necrotic and 454 normal TLs	aV40, rV40	5-year TLN incidence: aV40<5cc or rV40<10%, less than 5%; aV40≥10cc or rV40>15%, more than 20%.	rV40<10%; aV40<5 cc
Zhou et al. (33)	Case-controlled	86 patients, 53 necrotic and 119 normal TLs	V45	ORs increased with Dvs and Vds; V45 is predictive of TLN volume.	V45<15.1 cc
Feng et al. (34)	Cohort, retrospective	436 patients, 81 necrotic and 780 normal TLs	D2cc	TD5/5 for D2cc = 60.3 Gy; TD50/5 for D2cc = 76.9 Gy.	D2cc<60.3 Gy
Lu et al. (35)	Cohort, retrospective	188 patients, 94 necrotic and 282 normal TLs	V70	AUC for V70 in predicting TLN = 0.75.	V70<1.13 cc
Huang et al. (36)	Case-controlled	126 T4 patients, 83 necrotic and 169 normal TLs	D1cc, V20	D1cc >71.1 Gy *vs.* ≤71.1 Gy: OR for TLN, 7.92; V20 > 42.2 cc vs ≤42.2 cc: OR for TLN, 3.12.	D1cc<71.14 Gy; V20<42.2 cc
Gou et al. (37)	Cohort, retrospective	166 T3-4 patients, 22 necrotic and 310 normal TLs	Dmax	AUC for Dmax in predicting TLN = 0.861.	Dmax<78 Gy

TLN, temporal lobe necrosis; IMRT, intensity-modulated radiotherapy; AUC, area under receiver operating characteristic curve; LASSO, least absolute shrinkage and selection operator; TD, tolerance dose; TL, temporal lobe; OR, odd ratio; NA, not available.

Another plausible way to reduce the probability of CRN is based on stem cells. It has been previously demonstrated that radiation would weaken the reproductive capacity of O-2A progenitor cells (38–40) and eventually lead to CNS demyelination (41, 42). Accordingly, retransplantation of purified O-2A cells could remyelinate these lesions (43). Totipotent embryonic stem (ES) cells were also introduced as an unlimited donor for transplantation, given their self-renewing and multiple differentiation capacity. Brustle et al. found that transplantation of ES cells-derived precursors for oligodendrocytes and astrocytes could efficiently myelinated axons in CNS in a rat model with human myelin disease (44). Ijichi et al., through another *in vivo* study, suggested that the implantation of platelet derived growth factor (PDGF)-expressing cells increased O-2A progenitors in adult rat spinal cord without compromising their proliferation or differentiation potential (45). However, none of these strategies have ever been tested in patients with radiation necrosis, and future investigations are warranted.

## Conventional Management and Outcomes

It was once acknowledged that CRN represents a frequently irreversible and even progressive complication of radiotherapy (46), where conventional therapeutic approaches usually showed limited effectiveness. For decades, treatment strategy for CRN tended to be less aggressive, patients with asymptomatic CRN might be recommended to “wait and see,” while interventions were adopted mostly for those with typical symptoms or signs, including corticosteroids, anticoagulants, hyperbaric oxygen and surgery, etc. (**Table 3**) (47–54).

**Table 3:** Comparisons of conventional and novel treatment approaches for CRN.

Treatment	Medications	Evidence	Therapeutic effects			Adverse effects
			Edema	Necrosis	Clinical symptoms	
**Conventional treatment**						
Corticosteroids (47)	Methylprednisolone, prednisolone, dexamethasone	Cohort	Rapid, strong; Non-durable	Irreversible	Rapid remission of hypertension; weak effect on localized symptoms	Infection; osteoporosis; peptic ulcer; central obesity; hyperglucomia
Anticoagulants (48, 49)	Heparin, warfarin	Case series	Partial response	Irreversible	Minor-mild improvement on cerebral function	Bleeding
Hyperbaric oxygen (50–52)	NA	Case series	Partial response	Irreversible	Minor-mild improvement on cerebral function	Ear barotrauma, dyspnea
Surgery (53, 54),	NA	Case series	Instant, radical; Possibly relapse	Instant, radical; Possibly relapse	Instant remission of localized symptoms such as epilepsy; restoration of deteriorating cerebral function	Infection, bleeding, permanent neurological deficits, life-threatening in high-risk cases
**Novel treatment**						
anti-VEGF (55, 56),	Bevacizumab	RCT	Rapid, strong; Non-durable	Partly reversible	Rapid remission of hypertension and significant improvement on cerebral function	Bleeding, venous thrombosis, hypertension, aspiration pneumonia
Free radical scavengers (57)	Edaravone	RCT	Partial response	No additional improvement besides steroids	Significant improvement on neurologic symptoms and signs with LENT/SOMA scale	Insomnia, hyperglucomia, liver dysfunction
Gangliosides (58)	GM1	Preclinical	NA	NA	Neuroprotective effect on learning and memory impairment in rats with CRN	NA
NGF (59)	NGF	RCT	Rapid remission (with steroids)	High response rate, reversible	Durable remission of both hypertension and localized symptoms	Pain at the injection site

CRN, cerebral radiation necrosis; RCT, randomized clinical trial; LENT/SOMA, late effects normal tissue/subjective objective management analytic; NGF, nerve growth factor; GM1, monosialotetrahexosylganglioside; NA, not applicable.

### Management With Corticosteroids

A common practice for the treatment of CRN is using corticosteroids for necrosis-related edema. Dexamethasone usually produces prompt symptomatic relief in patients with focal RT necrosis and concomitant edema. Radiological improvement can also be found in certain cases receiving corticosteroids. However, this improvement is usually transient and steroid-dependent, leading to a rapid relapse once corticosteroids are stopped (47). Another concern was the potential risk of myriad debilitating chronic adverse effects with long-term use of corticosteroids, such as myopathy, endocrine and metabolic disorders, cardiovascular malfunctions, etc. In general, pulsed corticosteroid treatment would confer favorable response in terms of space occupying edema-related symptoms, but prolonged course and high-dose of corticosteroids should be given with special caution. Zhuo et al. reported that high-dose intravenous methylprednisolone is no superior to low-dose agent in treating CRN, thus recommending the use of corticosteroids as 1 mg/kg/day methylprednisolone for 5 days, then 40 mg for another 5 days, then oral prednisone for 30 mg/day initially, followed by gradual tapering by 5 mg/week to 10 mg/day. The maintenance period shouldn’t exceed 3 months (60).

### Management With Surgery

Surgical debulking of the necrotic brain tissue can provide helpful palliative effect for patients who fail to show adequate response to conservative treatments. Case series have shown that proper surgical intervention might rapidly ameliorate life-threatening intracranial hypertension and terminate inflammatory cascade reaction in brain tissue (53, 54). However, ample evidence also suggested that surgical intervention is not always necessary, for instance, symptoms will partially resolve with corticosteroid therapy alone in some cases, some necrotic lesions are inaccessible to surgery, and several focal necrosis would continue to deteriorate even after surgical debulking due to progressive necrosis near the original site (61, 62). In addition, gross total resection of necrotic debris has been demonstrated with no significant survival benefit when compared to conservative management (16).

### Management With Anticoagulants

Therapeutic anticoagulation has also been adopted to halt the progression of CRN based on a thought that CRN derives mainly from vascular damage-associated ischemia. Glantz et al. reported hopeful functional recovery in patients with CRN using heparin and warfarin anticoagulation (49). However, another case series found only modest efficacy of anticoagulation therapy on post-radiation neurotoxicity (48). Since these are only small size studies, solid conclusions can barely be drawn towards anticoagulation. Moreover, when using anticoagulants for CRN, one should take special caution that they might potentially cause bleeding, and the pros and cons should be weighed.

### Alternative Conventional Management Modalities

Hyperbaric oxygen therapy and high-dose vitamins were once proposed for symptomatic CRN (50–52). Up to date, however, these approaches have barely shown any potency in reversing cerebral necrosis, and no cases of complete resolution on both symptom and MRI abnormality have ever been reported.

## New Treatment Approaches for CRN

In recent years, with more understanding of the pathophysiology of CRN and the development of new drugs, some new management approaches using bevacizumab, nerve growth factor, gangliosides, and free radical scavengers have also found some striking results. A brief comparison of these agents is shown in **Table 3** (55–59).

### Treatment With Bevacizumab

Bevacizumab (Avastin), a humanized murine monoclonal antibody against the VEGF, has shown therapeutic effect in several solid tumors when used either alone or in combination with other cytotoxic drugs. Similar to tumor development in the vascular mechanism, CRN has been observed to response to Bevacizumab as well. Gonzalez first demonstrated that bevacizumab alone or combinatively could improve CRN-related edema with an underlying mechanism of normalizing the blood-brain barrier and reducing capillary leakage (55). As was widely accepted, VEGF overexpression is closely associated with radiation necrosis and subsequent brain edema (63), possibly by acting as a “vascular permeability factor” (64–66) that potently interrupts blood-brain barrier function and promotes vascular permeability. Therefore, blocking VEGF from approaching vascular targets could theoretically hinder fluid leak through capillary endothelium to the intercellular compartment, thus offering a plausible strategy for treating CRN.

Two retrospective studies have reported the experience of treating CRN with bevacizumab, one including six cases with histologically proven radionecrosis (67) and the other involving eight patients with MRI-based proof of CRN (55). Clinical stabilization or improvement and radiologic partial response and were observed in all cases. In a case report, a nearly complete response of the enhancement on MRI was observed after the use of bevacizumab (68), indicating that the process of CRN might be reversed. The first randomized placebo-controlled double-blind study that highlighted the role of bevacizumab in CRN was conducted in a small group of 14 patients by Levin et al. (69). After four doses of bevacizumab with 3-week interval, all patients in the treatment group showed improvement in neurologic symptoms or signs, compared to none in the control cases. Radiological evaluation on MRI scans showed that all patients treated with bevacizumab had a decrease in both the necrosis volume and the endothelial transfer constant. Tang et al. designed a larger-scaled randomized open-label study, in which 112 NPC patients with radiation brain necrosis were randomly assigned to receive bevacizumab or corticosteroid (56). This trial demonstrated a remarkable superiority of bevacizumab in not only improving edema and enhancement on MRI, but also neurological symptoms and cognitive function. However, the 6-month recurrence rate of CRN was similar between two groups, suggesting that bevacizumab might have limited efficacy in maintaining long-term response. Moreover, it should be noticed that bevacizumab is associated with certain toxicities. In Levin’s report, 6 of 11 patients receiving bevacizumab experienced adverse events, which included three serious cases with aspiration pneumonia, deep vein thrombosis-induced pulmonary embolism, and intracranial thrombosis. Tang reported an overall adverse events rate of 70.7%, in which most frequently seen was hypertension.

### Treatment With Nerve Growth Factor

Nerve growth factor (NGF) is one of the most prominent bioactive neurotrophic factors so far. NGF confers an important protective effect on central and peripheral nervous systems by preventing neural degeneration and promoting functional recovery of injured neurons (70). Given the evidence that radiation damage to oligodendrocytes and neurons is associated with late cerebral necrosis, we therefore postulated that NGF might be effective in treating cerebral radiation necrosis. The first case was treated with mouse NGF (mNGF) at 18 μg/injection per day for 2 months. Three months later, a cognitive improvement was observed with an increase from 25 to 30 in Folstein and Folstein Mini-Mental State Examination score, which persisted till the time of last follow-up 9 months later (71). mNGF also achieve a surprisingly complete response on MRI in this patient, featured by the disappearance of the Swiss cheese-like presentations in bilateral temporal lobes. To our knowledge, this was the first report indicating a therapeutic potency of NGF in CRN. Following this case, a prospective phase II clinical trial was conducted to test the efficacy of mNGF for symptomatic TLN. Fourteen patients were enrolled in this study. All patients had radiologically proven TLN following definitive RT for nasopharyngeal carcinoma and progressive neurologic symptoms or signs. mNGF combined with pulsed corticosteroids were prescribed for 2 months. Eight months later, contrast-enhanced MRI scans showed that five and seven patients respectively had complete response and partial response in the necrotic volume, only two patients didn’t respond to mNGF. Eight and five patients respectively showed complete and partial recovery in neurologic symptoms, while only one patient had no improvement. Adverse events were observed in three patients, all limited to mild injection site pain. This exploratory trial further demonstrated mNGF as a promising treatment option for TLN with minimal side effects (59).

### Treatment With Monosialotetrahexosylganglioside (GM1)

Gangliosides exist with large concentration in the central nervous system (CNS). As acidic glycolipids that constitute the major component of cell membranes, gangliosides play an important role in several neuronal events, such as augmenting neurite outgrowth, inducing neuron regeneration, and restoring impaired neural function (72). Exogenous administration of monosialotetrahexosylganglioside (GM1), a ganglioside also known as Sygen, showed that GM1 had an obvious effect on the nervous system by accelerating functional recovery of cholinergic and dopaminergic activities after injury, and by protecting neurons against retrograde degeneration (73, 74), thus indicating its potential effect in treating CNS diseases. These encouraging results have facilitated clinical trials in stroke and spinal cord injury. A placebo-controlled, double-blind randomized trial in 37 patients with spinal cord injuries showed that GM1 led to a significant improvement in American Spinal Injury Association (ASIA) motor score within 1-year follow-up (75). Another double-blind trial using GM1 in stroke also proved that gangliosides brought an improvement in neurologic scores that was more pronounced, more quickly and more persistent (76). Findings from another study show that GM1 ganglioside could powerfully ameliorate the cerebral edema in rats with mechanical lesion (77). Moreover, the function of GM1 in preventing retrograde degeneration and reducing the severity of behavioral effects after entorhinal lesions has also been reported (78). For CRN, *in vivo* experiments have also found a significant neuroprotective effect of GM1 on recovering learning and memory function in rats with radiation-induced brain injury (58). Although lack of clinical reports, GM1 has been applied in CRN across China for several years, with encouraging effect in ameliorating CRN-related symptoms. The common prescription is intravenous use of GM1 at 60 mg per day for 14 days, followed by 20 mg per day for at least 14 days. A few necrotic masses have been shown to almost completely resolve on MRI scans 3 months after the application of GM1(unpublished data). However, despite single-institutional experience, detailed data regarding the specific effect of GM1 in CRN remain scarce, thus calling for large, placebo controlled randomized studies for further investigation.

### Treatment With Free Radical Scavengers

As CRN is closely linked to intracranial oxidative stress, free radical scavengers such as Vitamin E and superoxide dismutase might theoretically benefit patients with CRN by eradicating oxygen-derived free radicals. Edaravone, a novel free radical scavenger, has been demonstrated *in vitro* to protect neurogenesis after RT by restoring human neural stem cells’ differentiation ability (79). In a prospective randomized clinical trial, edaravone provided significant improvement on MRI-detected edema as well as neurologic symptoms and signs (57). It would worth more trials to further elucidate the role of edaravone and other free radical scavengers in CRN.

## Conclusions

With a declining incidence owing to new radiotherapy technologies, TLN remains a remarkable complication in locally advanced NPC patients. Dosimetric prevention is the most important approach to manage TLN. Based on the accumulating knowledge in dose-volume effect, it has been proposed that both unnecessary “hot spot” and excessive radiation volume in TLs should be avoided. As for existing TLN lesions, traditional treatment modalities like steroids, anti-coagulants, and surgery have been unsatisfactory in either efficacy or safety. In comparison, newly applied medications, including bevacizumab, GM1, and nerve growth factor, etc, have shown potency in mitigating TLN both radiologically and symptomatically, and even completely reversing TLN lesions without serious side effects. More clinical trials should be encouraged in future to better explore these agents.

## Author Contributions

XZ and XW contributed to the design of the review as well as the manuscript drafting and revision. PL organized the literature search and contributed to the manuscript revision. All authors contributed to the article and approved the submitted version.

## Conflict of Interest

The authors declare that the research was conducted in the absence of any commercial or financial relationships that could be construed as a potential conflict of interest.
